# Mucopolysaccharidoses and the blood–brain barrier

**DOI:** 10.1186/s12987-022-00373-5

**Published:** 2022-09-19

**Authors:** Onur Sahin, Hannah P. Thompson, Grant W. Goodman, Jun Li, Akihiko Urayama

**Affiliations:** Department of Neurology, McGovern Medical School, University of Texas Health Science Center at Houston, Texas, USA

**Keywords:** Lysosomal storage disease, Mucopolysaccharidosis, Blood–brain barrier, Enzyme replacement therapy

## Abstract

Mucopolysaccharidoses comprise a set of genetic diseases marked by an enzymatic dysfunction in the degradation of glycosaminoglycans in lysosomes. There are eight clinically distinct types of mucopolysaccharidosis, some with various subtypes, based on which lysosomal enzyme is deficient and symptom severity. Patients with mucopolysaccharidosis can present with a variety of symptoms, including cognitive dysfunction, hepatosplenomegaly, skeletal abnormalities, and cardiopulmonary issues. Additionally, the onset and severity of symptoms can vary depending on the specific disorder, with symptoms typically arising during early childhood. While there is currently no cure for mucopolysaccharidosis, there are clinically approved therapies for the management of clinical symptoms, such as enzyme replacement therapy. Enzyme replacement therapy is typically administered intravenously, which allows for the systemic delivery of the deficient enzymes to peripheral organ sites. However, crossing the blood–brain barrier (BBB) to ameliorate the neurological symptoms of mucopolysaccharidosis continues to remain a challenge for these large macromolecules. In this review, we discuss the transport mechanisms for the delivery of lysosomal enzymes across the BBB. Additionally, we discuss the several therapeutic approaches, both preclinical and clinical, for the treatment of mucopolysaccharidoses.

## Introduction

Lysosomal storage diseases (LSDs) are autosomal recessive disorders characterized by an inherited deficiency in lysosomal metabolic activity. The lack of various acid hydrolases contributes to more than 50 different diseases. The prevalence of LSDs, as a group of disorders, is estimated as 1 in 5000–7000 live births [1, 2]. Importantly, over half of LSDs have central nervous system (CNS) involvement [3]. The neuropathology in LSDs is progressive and leads to premature death. While many LSD therapies, including enzyme replacements, ameliorate the storage of peripheral organs, treating the CNS remains an issue because of the blood–brain barrier (BBB) hampering the delivery of therapeutic drugs and biologics into the brain. Neurological deficits, including cognitive function and motor impairments, are early onset, debilitating symptoms of many LSDs. Once neurological deficits progress, reversing such impairments may be difficult as the relatively limited regeneration capacity of brain cells results in insufficient functional recovery. Thus, there is a pressing need to develop therapeutic interventions to treat CNS storage in LSDs. The transport of therapeutics across the BBB has been the major roadblock, not just for LSDs. The brain pathology of LSDs also overlaps with other neurodegenerative diseases, including Alzheimer’s and Parkinson’s diseases as the patients with Niemann-Pick type C exhibit neurofibrillary tangles commonly observed in Alzheimer’s disease patients [4–7], and the glucocerebrosidase gene mutation seen in Gaucher’s disease is also the major risk factor for Parkinson’s disease [8–10]. As such, treating LSDs lead to a high, unmet medical need and require the urgent development of therapeutic interventions, including improved enzyme replacement therapy (ERT) across the BBB.

Mucopolysaccharidosis (MPS), a subset of the LSDs, is characterized by the intracellular accumulation of glycosaminoglycans (GAGs). This GAG accumulation is due to a deficiency in the activity of lysosomal enzymes, which results in the inability of lysosomes to properly catabolize GAGs. Eventually, the buildup of these metabolic substrates can result in a wide range of somatic and neurological symptoms. For example, the accumulation of GAGs in growth plates and articular cartilage can increase chondrocyte apoptosis and inflammation, resulting in stunted growth, restricted range of motion in joints, and decreased mobility [11]. Additionally, cardiac manifestations due to arterial GAG deposition can be seen in the narrowing of arteries and reduced aortic elasticity of MPS patients [12]. There are also significant neurocognitive symptoms associated with MPS such as delayed development, behavioral impairment, and hydrocephalus [13]. MPS can be further categorized into individual subtypes based on the enzyme that is deficient and symptom severity.

These MPS types are summarized in Table 1 including the enzyme that is deficient, associated gene, accumulated substrate(s), disease onset, major symptoms, and currently available treatments. While clinical symptoms of MPSs are well summarized by Muenzer’s review: Overview of the Mucopolysaccharidoses, there is a subtype recently identified as MPS type X (MPS 10) [14]. MPS type X has been characterized by the deficiency of Glucuronate-2-sulfatase, also known as arylsulfatase K (ARSK). The enzyme ARSK, first identified by Wiegmann et al. is a lysosomal enzyme that hydrolyzes the sulfate esters moieties found on GAGs [15]. Due to the novelty of this finding, human patients eluded diagnosis until very recently when Verheyen et al. [16] located four patients from two different families in two different countries. Two variants of the enzyme ARSK deficiency were observed: a c.250C > T, p.(Arg84Cys) mutation seen in two children of a Turkish family, and a c.560T > A, p.(Leu187Ter) mutation in two children of an Indian family.

**Table 1:** Summary of traditional MPS types, characteristics, and available treatments

Name	Enzyme deficient	Gene	Accumulated substrate	Disease onset	Major symptoms	Currently available treatments
MPS I (Hurler/Scheie syndrome)	α-l-iduronidase	IDUA	Heparan sulfate Dermatan sulfate	Ages 1–15	Growth retardation, coarse face, hepatosplenomegaly, kyphosis, corneal clouding, heart valve abnormalities, hydrocephalus, hearing loss	ERT (IV) Eisengart et al., 2018; Shapiro et al., 2015; Wraith et al., 2007 [127–129] HSCT Aldenhoven et al., 2015; Shapiro et al., 2015; Eisengart et al., 2018 [127, 128, 130]
Hurler syndrome				Ages 1–2	Severe	
Hurler/Scheie syndrome				Ages 1–5	Intermediate	
Scheie syndrome				Ages 3–15	Mild	
MPS II (Hunter syndrome)	Iduronidase-2-sulfate	IDS	Heparan sulfate Dermatan sulfate	Ages 1–5	Mental/growth retardation, coarse face, hepatomegaly, kyphosis, hernias, skin abnormalities, heart valve abnormalities, spinal stenosis	ERT (IV) Kim et al., 2017 [131]
MPS III (Sanfilippo syndrome)			Heparan sulfate	Ages 1–4	Mental retardation, CNS degeneration, development delay, coarse face, hepatosplenomegaly, seizures, hyperactivity, aggression	No approved treatment Kong et al., 2020 [132]
IIIA	Heparan-*N*-sulfatase	SGSH				
IIIB	α-*N*-acetylglucosamine 6-sulfatase	NAGLU				
IIIC	α-glucosaminidase acetyltransferase	HGSNAT				
IIID	*N*-acetylglucosamine 6-sulfatase	GNS				
MPS IV (Morquio syndrome)			Keratan sulfate	Ages 2–4	Coarse face, skeletal abnormalities, hearing loss, corneal clouding	─
IVA	*N*-Acetylgalactoasmine-6-sulfate sulfatase	GALNS			Severe	ERT (IV) & HSCT Sawamoto et al., 2020 [133]
IVB	β-galactosidase	GLB1			Mild	─
MPS VI (Maroteaux-Lamy syndrome)	*N*-acetylgalactosamine 4-sulfatase	ARSB	Dermatan sulfate	Ages 2–5	Coarse face, umbilical hernia, hepatosplenomegaly, corneal clouding, skeletal abnormalities	ERT (IV) D'Avanzo et al., 2021 [134] HSCT Turbeville et al., 2011 [135]
MPS VII (Sly syndrome)	β-glucuronidase	GUSB	Heparan sulfate Dermatan sulfate	Birth-year 1	Hydrops fetalis, Hernias, short stature, heart disease, coarse face, hydrocephalus, corneal clouding, hepatosplenomegaly, cognitive impairment	ERT (IV) Parini and Deodato, 2020 [40] HSCT Taylor et al., 2019 [136]
MPS IX (Natowicz syndrome)	Hyaluronidase	HYAL1	Hyaluronan	Birth-year 1	Cleft palate, development of soft tissue masses, short stature, hyperplasia	─
MPS X	Glucuronate-2-sulfatase (Arylsulfatase K)	ARSK	Heparan sulfate Chondroitin sulfate Dermatan sulfate	─	Coarse facial features; Skeletal, vision, and cardiac abnormalities	─

─ Data unavailable

While both variants had skeletal abnormalities, including short trunk statures, genu valgum, and coarse facial features, the two variants had some differences. For example, individuals with the c.250C > T mutation had cardiac and mild ocular abnormalities, whereas one of the individuals with a c.560T > A presented with brachydactyly and renal calculi [16]. More investigation is necessary as the sample size is limited preventing further characterization of symptom presentation in MPS type X patients. The characteristics of this subtype have been corroborated by findings in an ARSK knock-out mouse model [17]. This study demonstrated chondroitin and heparan sulfate accumulation suggesting the enzyme ARSK plays a significant role in lysosomal clearance. The main difference between the murine model and humans is that the mice lack the skeletal abnormalities seen so far in the affected human patients of MPS type X.

MPS IIIE is an additional new subtype which results from a deficiency of the Arylsulfatase G (*N*-glucosamine 3-*O* sulfatase; ARSG). However, this new subtype of MPS has only recently been discovered in a study conducting a genetic knockout in the ARSG gene associated with these enzymes and has yet to be recorded in a human case [18]. While Usher disease which is caused by the mutation(s) of ARSG has shown MPS-like symptoms, the clarification if MPSIIIE and Usher disease are identical awaits the identification of the location and type of mutations in ARSG [17, 19].

While new subtype(s) of MPS are being characterized, the treatment options for MPSs have been limited. There is a pressing need to overcome the CNS symptoms which could be achieved by developing effective BBB transport. Apart from its clinical significance, one of the primary interests in the research related to MPS is in its value as an ideal disease model for testing trans-BBB therapeutic strategies. The advantages of the MPS models for developing brain delivery approaches include; (i) MPS is a monogenic disorder. The cause of the disease is due solely to the lack or mutation of a specific lysosomal enzyme, (ii) a lysosomal enzyme is a high-affinity physiologic ligand for the cation-independent mannose 6 phosphate receptor (CI-MPR) which is ubiquitously expressed in many brain cells, so the binding and release of the lysosomal enzyme is inherently controlled, (iii) small amounts of delivery are therapeutic, as increasing levels of the lysosomal enzyme by only 1–3% [20] is believed to be sufficient to improve outcomes, (iv) peripheral organ uptake is a benefit, not a side effect, because of systemic deficiency of a specific enzyme in each MPS, and (v) there are no concerns of biodegradation of lysosomal enzymes as they work in lysosomes, which is the target location for the delivery. In addition to these advantages in employing MPS as a disease model to develop a new BBB delivery strategy, most of the lysosomal enzymes share the common feature that the M6P moiety at the end of glycans allows them to bind to the CI-MPR.

This makes the delivery strategy a more universal approach to treating neurodegenerative lysosomal diseases, which affect many children worldwide. Table 2 summarizes the prevalence of MPS in various countries per 100,000 live births. MPS is a rare disease with the prevalence ranging from 0.00071 for MPS III in the United States to 1.89 in the Netherlands per 100,000 live births [21, 22]. Additionally, some subtypes of MPS such as MPS IX are so rare that data were either unable to be collected or no cases were reported (Table 2). Also, MPS X was not included in the presented meta-analyses due to its recent identification. Due to the rarity and complexity of the disease, there are several difficulties in the identification of these MPS cases. These limitations include the variations of disease severity and onset within the subcategories as well as the limited number of documented cases [13]. As a result of recent developments and investigations, additional large-scale and up-to-date analyses are necessary to further investigate global prevalence.

**Table 2:** Prevalence rates of MPS in various countries per 100,000 live births

	Prevalence per 100,000 live births											
Country Study period	Japan 1982–2009	Switzerland 1982–2009	United States 1995–2005	Poland 1970–2010	Brazil 1994–2018	The Netherlands 1970–1996	Australia 1980–1996	Northern Portugal 1982–2001	Czech Republic 1975–2008	Sweden 1975–2004	Denmark 1975–2004	Norway 1979–2004
References	Khan et al., 2017 [137]		Puckett et al., 2021 [21]	Jurecka et al., 2014 [138]	Josahkian et al., 2021 [139]	Poorthuis et al., 1999 [22]	Meikle et al., 1999 [1]	Pinto et al., 2004 [140]	Poupetová et al., 2010 [141]	Malm et al., 2008 [142]		
MPS I	0.23	0.19	0.0007	0.22	0.29	1.19	1.14	─	0.72	0.67	0.54	1.85
Hurler								1.05	─			
Hurler/Scheie								1.33	─			
Scheie								0.28	─			
MPS II	0.84	0.46	0.0007	0.46	0.48	0.67	0.74	1.09	0.43	0.27	0.27	0.13
MPS III	0.26	0.38	0.00071	0.86	─	1.89	1.51	0.84	0.91	0.67	0.43	0.27
IIIA	─	─	0.00052	─	0.08	1.16	─	0	0.47	─	─	─
IIIB	─	─	0.00014	─	0.12	0.42	─	0.72	0.02	─	─	─
IIIC	─	─	0.00004	─	0.07	0.21	─	0.12	0.42	─	─	─
IIID	─	─	0	─	0.001	0.1	─	─	0	─	─	─
MPS IV	0.15	0.38	0.00038	0.14	─	─	0.59	─	0.73	0.07	0.48	0.76
IVA	─	─	0.00029	─	0.15	0.22	─	0.6	0.71	─	─	─
IVB	─	─	0.00001	─	0.003	0.14	─	─	0.02	─	─	─
MPS VI	0.03	0.11	0.00011	0.0132	0.35	0.15	0.43	0.42	0.05	0.07	0.05	0.07
MPS VII	0.02	0.038	0.00007	0	0.02	0.24	0.047	0	0.02	─	─	─
MPS IX	─	─	0	0	0	─	─	─	─	─	─	─
MPS X	─											

─ Data unavailable

## Current therapeutic approaches to MPSs

While there is no curative therapy for MPSs, there are several approaches targeting the clinical manifestations secondary to the disease. MPS patients commonly have mental retardation, organomegaly, multiple skeletal anomalies referred to as dysostosis multiplex, coarse facial features, corneal clouding, hearing impairment, increased urinary GAGs, widespread lysosomal storage of GAGs in most tissues, including the CNS, and premature death [14, 23]. Currently studied therapies include hematopoietic stem cell therapy (HSCT), chaperone therapy (CT), substrate reduction therapy (SRT), gene therapy, and ERT. Although significant progress has been made on these approaches, clinically approved therapies for MPSs were currently limited to HSCT and ERTs (Table 1) [24]. However, two SRT agents are approved for treating type I Gaucher’s disease, another type of LSDs, while treating the CNS symptoms remains an issue [25–27].

HSCT relies on the ability of the donor stem cells to partially replace the patient's microglia permitting the delivery of the deficient enzyme to the neurons [28]. HSCT has been considered the standard treatment option for MPS I–Hurler syndrome due to the demonstrated preservation of cognitive function in children [29]. Notably, the patients’ outcomes following this method can be predicted based on the age of the recipient receiving the transplantation as well as the enzyme production of the graft itself [30]. Drawbacks of this methodology include the necessity of early diagnosis to improve outcomes, delays in efficacy speed, and possible risk of graft rejection.

CT and SRT have been explored for the treatment of MPS. CT involves pharmacological molecules that bind to unstable enzymes in a selective manner to improve enzymatic functions. Preventing the mutation-associated misfolding of a lysosomal enzyme is beneficial for treating LSDs that are associated with enzyme mutants that are biologically active but intrinsically unstable. In CT, a small bioactive chaperone molecule is given intravenously to assist in the proper folding of lysosomal enzyme deficiencies caused by conformational changes [31]. CT has been explored for the treatment of MPS II, IVA and IVB [32] and has been given in combination with other therapeutic options. While CT has some advantages, such as the lack of immunogenicity, not all MPS subtypes are due to the conformational misfolding caused by missense mutations, which limits the scope of MPS diseases that can be treated by CT [31]. Conversely, while other therapeutic methods focus on increasing the enzymatic activity to overcome lysosomal deficiencies, the basis for SRT is to reduce the synthesis of substrates upstream from the dysfunctional degradation step [33]. By decreasing the amount of upstream substrate, the buildup of the undegraded products can be mitigated. While potential lack of specificity in target upstream substrates, SRTs have been explored for MPS I, II and VI [34].

ERT is considered one of the most straightforward approaches to treating LSDs. ERT refers to the treatment of inherited deficiency of a lysosomal enzyme by administering a specific enzyme missed in LSDs. ERT reduces visceral lysosomal storage, and normalizes the pathological phenotype in peripheral organs. One of the two clinically approved therapeutic options in MPS treatment, ERT was initially developed by Barton et al. (1991) as a treatment for Gaucher’s disease and, following its success, has since been adapted to treat a number of MPS subtypes, including MPS I, MPS II, MPS IVA, MPS VI, and MPS VII [20, 35–40]. Fundamentally, ERT consists of semi-weekly intravenous infusions of the deficient lysosomal enzyme. ERT with murine or human lysosomal enzymes has been shown to reduce visceral lysosomal storage, normalize the pathological phenotype, and prolong lifespan in rodent models [41–43]. It also has shown improvements in abnormal storage of lysosomal metabolites in the brain if treatment is begun before 2 weeks of age [44]. However, correcting impairments of the lysosomal clearance in the CNS has been challenging due to the presence of the BBB, which hampers the entry of circulating lysosomal enzymes into the brain [23, 45].

In our previous studies, we found that CI-MPR transported lysosomal enzymes containing M6P moieties across the BBB [46, 47]. This receptor-mediated transport of lysosomal enzymes showed developmental down-regulation that resulted in a failure of delivery of lysosomal enzymes across the BBB in the adult brain [46, 47]. One of the main disadvantages of this therapy is that the adult BBB, unlike the neonatal one, does not transport lysosomal enzymes into the brain, making ERT ineffective in treating the CNS symptoms of LSDs. Another notable disadvantage of ERT is the development of antibodies against the infused enzyme which can result in an undesirable immunological response. However, this response varies widely depending on the MPS treatment [48].

While the complexity of mechanisms that regulate the BBB, the transcytosis of compounds, including small molecule drugs and biologics, across the BBB is an ongoing field of research with many questions still unanswered. Thus, the following section will discuss the current literature that explores the underlying mechanisms that regulate transcytosis across the BBB.

## BBB transport

**Fig. 1:**
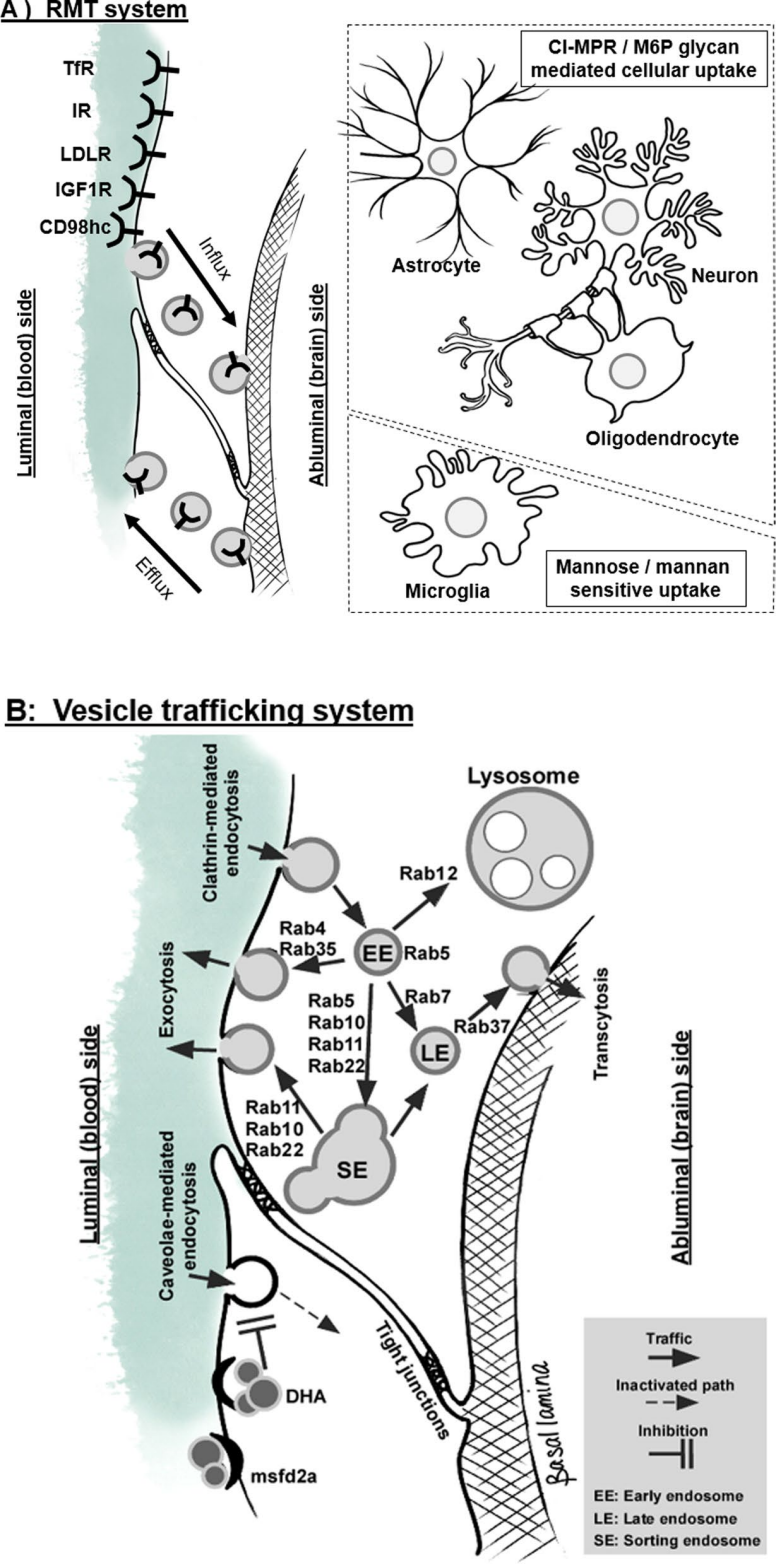
Schematic representation of the RMT system and endosomal trafficking mechanisms in the brain endothelial cells. **A** Innate RMT systems available at the BBB. The cellular uptake of lysosomal enzymes in brain cells were mostly by CI-MPR in neurons, astrocytes, and oligodendrocytes. However, microglia show mannose and mannan mediations. TfR: Transferrin receptor; IR: Insulin receptor; LDLR: low-density lipoprotein receptor; insulin-like growth factor 1 receptor. **B** Intracellular vesicle trafficking and Rab small GTPases

The BBB is the interface between the CNS and the cerebrovascular system. This complex neurovascular structure is composed of a single layer of brain microvessel endothelial cells, surrounded by basal lamina, astrocytic endfoot, pericytes, and some direct projection of neurons [49, 50]. Tight junctions expressed between the endothelial cells regulate the free exchange of solutes in both blood-to-brain and brain-to-blood directions by sealing the endothelial junctional clefts which are synergistically regulated by adherens junctions. Both transcellular and paracellular routes are important pathways for the delivery of CNS drugs while the regulatory mechanisms for these pathways are distinctively different from each other (Fig. 1). While paracellular transport across the tight junctions is normally limited to hydrated ions [51], ultrasound-induced microbubble cavitation can stretch these junctions to allow for the passage of compounds up to 70 kDa [52].

Transcellular transport across the BBB is regulated by multiple mechanisms, mainly including clathrin- and caveolae-mediated endocytoses. Once vesicles were formed and endocytosed from the plasma membrane of endothelial cells, their intracellular trafficking is regulated by guidance molecules such as small GTPases called Rab proteins. Also, recent studies demonstrated how caveolae-mediated endocytosis is limited at the brain endothelial cells forming the BBB. Ben-Zvi et al. [53] found the critical role of the major facilitator superfamily domain containing 2a (Mfsd2a) in enhancing the integrity of the BBB in concert with pericyte-mediated regulations. Genetic ablation of Mfsd2a induced extensive formation of transcytotic vesicles [53]. Such vesicular transcytosis mediated by caveolae was constitutively inhibited by Mfsd2a-transported lipids on the endothelial cell surface membrane [54]. Also, a recent study suggested an extracellular matrix component secreted from pericytes plays a role in regulating endocytosis by potentially keeping proper mechanical stimuli on brain endothelial cells [55]. While these recent advances substantially increased our understanding of the regulatory mechanisms of barrier permeability, the inability to deliver a lysosomal enzyme across the BBB has been a major challenge.

Several approaches to delivering lysosomal enzymes across the adult BBB have been developed, including bioengineered lysosomal enzymes with a cell-permeable peptide modification, enzymes tagged with other receptor recognition motifs, and re-induction of CI-MPR at the surface of BBB. These approaches collectively employ endogenous features of brain endothelial cells [23, 56] including the adsorptive-mediated transport from the plasma membrane, alternative receptors constitutively expressed at the luminal surface of the endothelial cells, and pharmacologic manipulation of endocytic pathways, respectively. Intracerebroventricular (ICV) injection of lysosomal enzymes has also been studied to bypass the complexity of BBB transport, taking advantage of the brain entry of cerebrospinal fluid containing the enzyme molecule through the perivascular space for the purpose of delivery. In the following section, we discuss the receptor-mediated transport (RMT) systems being studied for brain delivery and intra-endothelial cell trafficking mechanisms.

## Receptor-mediated transport for brain drug delivery

### Transferrin receptor

The brain delivery of biologics using the RMT processes has long been studied by making a drug conjugation to receptor ligands of RMT across the BBB [56–60]. There are several RMT processes exist at the BBB, including the low-density lipoprotein receptor, insulin-like growth factor receptor, insulin receptor, and transferrin receptor (TfR) [58, 60–63]. The latter two RMT systems are the ones most extensively studied for the delivery of lysosomal enzymes across the BBB [59, 60, 62, 64–66]. Their cellular uptake processes are driven by the clathrin-mediated endocytotic process of the receptors available at the luminal surface of the brain endothelial cells.

The enriched expression of TfR at the brain endothelial cells made TfR a suitable target for the RMT of therapeutics, especially that applies to the delivery of biological macromolecules [61, 67]. An earlier study found that TfR undergoes endocytosis in a ligand-independent manner [68] Also, TfR has a large capacity to deliver bioconjugated macromolecules across the BBB mediated through the antibody directed to the receptor [69]. There are many approaches targeting TfR have been explored which are summarized in the review by Terstappen et al. [56].

Recent studies employed contemporary bioengineered approaches including the use of bispecific antibodies [70, 71], and Fc domain bivalent antibodies [65, 66, 72]. TfR exerts a bidirectional transport which refers to both influx (blood-to-brain) and efflux (brain-to-blood) directions. Thus, to effectively deliver therapeutic molecules across the BBB, it is essential to release the therapeutics at the abluminal side of the BBB after the transcytosis in the brain endothelium. For example, having a higher affinity motif to bind TfR will efficiently recognize the luminally expressed TfR to endocytose the therapeutics; however, the release of therapeutics after crossing the BBB may be insufficient due to the high-affinity binding to the receptor.

Eventually, Bien-Ly et al. showed that whereas high-affinity antibodies for TfR (20 nM) were trafficked to lysosomes and eventually degraded, lower affinity antibodies for TfR (600 nM) had increased delivery into the brain [73]. Additionally, Sade et al. showed that antibodies with decreased affinities for TfR at lysosomal pHs near 5.5 compared to antibodies with constant affinity regardless of pH had increased transcytosis and avoided lysosomal degradation in an in vitro model, suggesting that the dissociation of the antibody-receptor complex is as important as antibody-receptor affinity [74]. In addition to these findings, a series of recent studies indicate that a lysosomal enzyme fused to an antibody fragment with a moderate affinity to TfR is distributed in the brain to a greater extent compared to the conjugate with a high-affinity motif [65, 66], confirming the importance of binding affinity for innate receptors used for BBB transport.

Also, the effective delivery of macromolecule therapeutics may depend on the levels of target receptors available for RMT at the BBB with respect to development, aging, and disease conditions. While the recent study suggested the expression of TfR at the BBB showed an age-dependent decrease [75], the previous studies found the levels of TfR remain unchanged with age and other neurodegenerative conditions including murine Alzheimer’s disease models and AD patients [76, 77]. It is uncertain if LSDs impact the receptors at the BBB and endocytic vesicle trafficking, further study will warrant the aging and disease-specific RMT approach.

Furthermore, in addition to the affinity and the availability of innate RMT systems, the bidirectional nature of transcellular transport may need to manage for effective delivery. As evidence suggests [58–60, 78], transcytosis across the BBB is a bi-directional process. While TfR exerts ligand-independent transcytosis in brain endothelial cells [68], any conventional antibody targeting TfR, regardless of its affinity, behaves indistinguishably from endogenous ligand transferrin in terms of bi-directional transportation. This inevitably leads to a concentration equilibrium of the antibody between the two sides separated by the BBB. Thus, developing a strategy that breaks the balance of bi-directional transportation to enhance the blood-to-brain directionality may be the key to the further effective delivery of therapeutics across the BBB.

Further alternative approaches include the exploration of new RMT systems. While TfR, was extensively explored for RMT, recent studies have identified potentially new receptors that could be used, such as CD98hc. CD98hc is a heavy chain subunit of the heterodimeric LAT1 membrane transport proteins [79, 80], which are overexpressed in brain endothelial cells making them a promising target for the preferential delivery of enzymes to the brain. Though CD98hc has not yet been explored as a delivery method for ERT in MPSs, Zuchero et al. have shown CD98hc is highly enriched in the brain endothelial cells and the antibody directed to CD98hc successfully increased the transport across the BBB in vivo, suggesting the potential of CD98hc for macromolecule delivery [80].

### CI-MPR

Another important biomolecule involved in transcytosis, M6P glycan is a molecular tag found on lysosomal enzymes that signals their delivery from the Golgi network to the lysosome. Additionally, the CI-MPR, which is found on the plasma membrane of cells, is used to uptake lysosomal enzymes found in the extracellular space after crossing the BBB, and deliver them to the lysosome. In addition to the role of the CI-MPR in extracellular uptake, recent studies have also explored the use of the CI-MPR in crossing the BBB. Siupka et al. demonstrated that the CI-MPR can undergo bidirectional trafficking between luminal and abluminal membranes through Vps35-positive vesicles in bovine and porcine brain endothelial cells, suggesting that it could be a potential target for RMT [81].

Urayama et al. tested the efficacy of M6P glycan targeting in ERT using phosphorylated and non-phosphorylated beta-glucuronidase (GUSB) to treat neonatal and adult MPS VII mice [46]. Whereas non-phosphorylated GUSB was not absorbed well in either neonatal or adult mice, interestingly, phosphorylated GUSB was taken up in neonatal mice, but not adult mice. These findings indicate that the CI-MPR expression in the brain endothelium was downregulated during development. A similar experiment investigating RMT showed the successful delivery of sulfamidase into the brain of neonatal, but not adult, MPS IIIA mice, suggesting that the CI-MPR could be used as a general lysosomal enzyme delivery target across the BBB in neonates [46]. Additionally, Matthes et al. showed that recombinant human arylsulfatase A can be taken up for transcellular transport across the BBB in a metachromatic leukodystrophy mouse model by either decreasing the negative charge of the enzyme for adsorptive transcytosis, or by RMT via CI-MPR through increasing the number of M6P glycan on the enzyme [82]. To expand the efficacy of the CI-MPR targeting beyond neonates, in vivo murine studies have shown that enzyme delivery with adjunctive adrenergic agonists can increase the delivery of ERTs to the brain via the CI-MPR. Recombinant human acid α-glucosidase administered with either albuterol or clenbuterol in mice with Pompe disease showed an increase in ERT efficacy when compared to controls in both skeletal muscle and brain tissue due to an increase in CI-MPR expression (83).

Recently, the remodeling of lysosomal enzyme, recombinant human acid α-glucosidase with high-affinity M6P glycan enhanced the binding affinity to CI-MPR and cellular uptake [84]. While this approach was not tested in the context of LSDs including the mouse model of Pompe disease, a lysosomal enzyme with remodeled M6P glycans may increase the therapeutic efficacy of ERTs. Also, intracerebroventricular ERT was explored with β-galactosidase [85]. In a mouse model of GM1 gangliosidosis, injected recombinant human β-galactosidase was taken up by brain cells through CI-MPR, substantially reduced CNS storage and reversed neuropathology, indicating the availability of CI-MPR mediated uptake mechanism in brain parenchymal cells once a lysosomal enzyme is delivered into the brain across the BBB where the CI-MPR is developmentally downregulated.

Regardless of brain delivery approaches, lysosomal enzymes need to be located in lysosomes in brain cells to exert their function. A recent insightful study with arylsulfatase A (ASA) found that the uptake rate of ASA showed a brain cell dependency [86]. While CI-MPR mediated the uptake of ASA in primary cultured neurons, astrocytes, and oligodendrocytes, the uptake in microglia was insensitive to M6P inhibition, but mannose and mannan partially inhibited the cellular uptake of ASA, suggesting that CI-MPR is not the major uptake mechanism in microglia. While CI-MPR is ubiquitously expressed in mammalian cells, its availability on the plasma membrane of cells for lysosomal enzyme delivery may vary. Thus, these results also suggest the necessity of the development of a cell type-specific delivery approach to comprehensively treat the CNS storage of LSDs.

### MFSD2A

MFSD2a, previously an orphan receptor, is now known to act as both a key regulator of the BBB [53], while also functioning to transport docosahexaenoic acid (DHA), an omega-3 fatty acid, into the brain [87]. Due to the exclusive expression of MFSD2a on brain endothelial cells, MFSD2a could be a promising new target for manipulating macromolecule transport across the BBB. However, one potential challenge to this strategy is the dual role of MFSD2a, including the uptake of DHA to enrich it at the plasma membrane of brain endothelial cells, and at the same time, its inhibitory regulation of caveolae-mediated transport resulted in decreased transcytosis across the BBB [88]. As shown by Wang increased Wnt signaling resulted in a corresponding increase in MFSD2a expression, but also a decrease in caveolin-1 mediated transcytosis across the vascular endothelium in the blood-retinal barrier [89] supporting the role of MFSD2a for maintaining the integrity of the barrier.

However, two strategies were recently proposed to take advantage of this duality in MFSD2a [90]. The first strategy was based on MFSD2a inhibition, allowing for an increase in caveolin-mediated transcytosis, while the second strategy involved the conjugated of therapeutics to DHA to allow for the lipid-drug conjugated to be transported across the BBB by MFSD2a. This first strategy of MFSD2a inhibition was recently realized by Ju where tunicamycin conjugated nanoparticles inhibited MFSD2a activity for the transport of chemotherapy into a murine brain [91]. While this approach was used to treat breast metastases in the brain, a similar approach could be used as a delivery strategy for treating CNS symptoms of lysosomal storage diseases including MPSs.

## Intracellular trafficking in brain endothelial cells

In RMT, once the receptor-ligand enzyme complex progressed for the internalization from the plasma membrane of brain endothelial cells, the vesicular transport receives directional regulations via Rab small GTPases. Figure 1 and Table 3 summarizes how intracellular vesicle trafficking is coordinated by Rab proteins. Rab5 is a key regulator of endosome fusion and trafficking to early endosomes where the endosome further progresses to sorting endosomes by Rab 5, 10, 11, and 22. The early recycling of internalized receptors to the plasma membrane is regulated by Rab 4 and 35. Rab 11 mediates the function of the recycling endosome in directing vesicle traffic to the cell surface plasma membrane where receptors such as TfR are relocated. Rabs 10 and 22 also participate in this recycling process. Also, it is known that internalized TfR in endosomes is guided by Rab12 resulting in lysosomal degradation. Transmembrane transport is mediated through the guidance by Rab 35 and 37, thereby endosomal components cross the full width of brain endothelial cells.

**Table 3:** Summary of previous studies showing the expression of the function of Rab proteins in non-brain and brain endothelial cells

Rab GTPases	Non-brain endothelial cells	Brain endothelial cells
Rab4 Early recycling	Sluijs et al., 1992 [143] Roberts et al., 2001 [144] Schnatwinkel et al., 2004 [145]	Ward et al., 2005 [146] Villaseñor et al., 2017 [95]
Rab5 Early endosome Sorting endosome	Bucci et al., 1992 [147]	Villaseñor et al., 2017 [95] Tian et al., 2020 [96]
Rab7 Late endosome	Vanlandingham and Ceresa, 2009 [148] Girard et al., 2014 [149] Zhang et al., 2009 [150]	Villaseñor et al., 2017 [95] Tian et al., 2020 [96] Alam et al., 2020 [97]
Rab10 Recycling path	Babbey et al., 2006 [151]	Gross et al., 2021 [152]
Rab11 Recycling path	Ullrich et al., 1996 [153] Lock and Stow, 2005 [154]	Ward et al., 2005 [146] Tian et al., 2020 [96]
Rab12 Lysosomal sorting	Matsui et al., 2011 [155] Matsui and Fukuda, 2011 [156]	–
Rab17 Transcytosis	Lütcke et al., 1993 [92]	Not expressed Villaseñor et al., 2017 [95] Zhang et al., 2014 [94]
Rab 22a Sorting endosome Recycling path	Magadán et al., 2006 [157] Zhu et al., 2009 [158]	Villaseñor et al., 2017 [95]
Rab25 Transcytosis	Tzaban et al., 2009 [93]	Not expressed Villaseñor et al., 2017 [95] Zhang et al., 2014 [94]
Rab35 Recycling	Mrozowska and Fukuda, 2016 [159]	Biesemann et al., 2017 [160]
Rab 37 Transcytosis	Tzeng et al., 2018 [161]	Zografou et al., 2012 [162]

─ Data unavailable

These fine regulations of endosomal vesicle trafficking mechanism were historically studied in non-brain endothelial cells, such as Hela epithelial cells, Chinese hamster ovary cells, fibroblast, etc. Table 3 summarizes the functional expression of Rab proteins in brain endothelial cells in relation to endosomal trafficking and sorting tubule formation. Interestingly, Rab 17 and 25, well-established transcytosis regulators in epithelial cells [92, 93], were little expressed in brain endothelial cells [94, 95] which suggests the cell-type specificity in the regulatory manner of Rab GTPases. Further study is necessary to have a comprehensive understanding of the vesicle trafficking mechanism and its regulation in the BBB. As a compelling recent study found that Rab 7, a late endosomal sorting marker [96], was postulated also as a transcytosis marker in a-synuclein transport across the BBB [97]. This implies the roles of Rab proteins may show a pathological context dependency which may have a considerable impact on the effective delivery of therapeutics across the BBB.

Currently, it is unfortunate that our knowledge of intracellular trafficking in MPS-related endothelial cells is very limited. Thus, it is essential to investigate if abnormal lysosomal storage alters the intracellular trafficking mechanisms which may fundamentally be of importance for successful transcellular transport of therapeutics across the BBB underlie in a pathological context of LSDs. Though there have been significant advances in recent years in understanding the mechanisms that regulate crossing the BBB, many open questions remain. For example, cerebral vasculature is zonated and shows unique cellular profiles and functionalities [98, 99]. The part of the vasculature (arterioles, capillaries, or venules) that is involved in the transport across the BBB is understudied. Kucharz et al. showed that receptor-mediated transcytosis via TfR primarily occurs at post-capillary venules due to the perivascular space, which allows for less diffusion resistance [100]. However, it is unknown whether these findings apply to other receptors such as insulin receptor, insulin-like growth factor receptor-1, low-density lipoprotein receptor (LDLR), or CD98hc, which have been attempted to utilize for BBB transport [60, 63, 80, 101].

Another open question is the differences in brain endothelium in murine models versus clinical patients in assessing the predictive efficacy of RMT delivery methods. Zhang et al. extracted RNA from human and murine brain samples as well as collected public RNA-seq data to show that there was a significant increase in TfR, LDLR-related protein 1, Insulin-like growth factor-1 receptor (IGF1R) expression in murine brain vasculature compared to human brain vasculature, highlighting the shortcomings in murine models as a clinical predictor of BBB delivery methods [102].

## Preclinical advancements in MPS therapies

In recent years, there have been novel preclinical research investigating the delivery of enzymes across the BBB for the treatment of various types of MPS (Table 4). Sonada et al. conjugated a novel fusion protein complex to treat MPS II by combining iduronate-2-sulfatase (IDS) with an anti-TfR antibody, and demonstrated the proof-of-concept efficacy of their enzyme-antibody complex in reducing GAG accumulation in the brain parenchyma and peripheral tissue of an MPS II mouse model, though they did not test neurocognitive function of treated mice in the study [64]. More recently, this group tested their novel complex, termed pabinafusp alfa, again in a murine MPS II model. The results of their study showed not only a decrease in heparan sulfate concentration in the brain tissue of treated groups, but also a potential protective effect against the neurodegeneration associated with MPS II, as measured via the Morris water maze test [103].

**Table 4:** Summary of MPS pre-clinical enzyme replacement therapy research with focus on the CNS

MPS Type	Lysosomal Enzyme	Modification	Administration	Effect in brain	Animal model	References
MPS I	α-l-iduronidase	IgG fusion (TfR)	IV	CNS storage reduced	MPS-I cats	Boado et al., 2011 [163]
		AAV encoded (AAV8-MCI)	IN	Enzyme activity found	MPS-I mice	Wolf et al., 2012 [164]
		ApoE motif conjugated	Hepatic expression	CNS storage reduced	MPS-I mice	Wang et al., 2013 [165]
		IgG fusion(HIR)	IV	Brain distribution	Rhesus monkey	Boado and Pardridge., 2017 [166]
		AAV encoded (AAV9)	IN	CNS storage reduced; Increased enzyme activity	MPS-I mice	Belur et al., 2017 [167]
		RTB fusion	IV	CNS storage reduced; Increased enzyme activity; improved neurocognition	MPS-I mice	Ou et al., 2018 [107]
		AAV encoded (AAV9)	ICV	Enzyme activity found (widespread)	MPS-I mice	Belur et al., 2021 [168]
			IT	Enzyme activity found (scattered, mostly hindbrain)		
			IN	Enzyme activity found (exclusively olfactory bulb)		
MPS II	Iduronidase-2-sulfate	─	IV (high dose)	CNS storage reduced	MPS-II mice	Polito et al., 2010 [169]
		─	ICV	CNS storage reduced	MPS-II mice	Higuchi et al., 2012 [170]
		─	ICV, IT	Brain distribution	Cynomolgus monkeys	Calias et al., 2012 [171]
					Beagle dogs	
			IT	CNS storage reduced	MPS-II mice	
		─	IV	CNS storage reduced; Maintained neurocognitive status	MPS-II mice	Cho et al., 2015 [108]
		IgG fusion	IV	CNS storage reduced; Brain distribution	MPS-II mice	Sonoda et al., 2018 [64]
					Cynomolgus monkeys	
		Encapsulated in nanoparticles	IV	CNS storage reduced; reduction of neuro and inflammatory markers	MPS II mice	Rigon et al., 2019 [106]
		ETV fusion	IV	CNS storage reduced; Brain distribution	MPS-II mice	Ullman et al., 2020 [65]
		Recombinant human fusion	IV	CNS storage reduced; Maintained neurocognitive status	MPS-II mice	Morimoto et al., 2021 [103]
MPS IIIA	Heparan-*N*-sulfatase	Chemical	IV (high dose)	Minor brain distribution	MPS-IIIA mice	Rozaklis et al., 2011 [172]
		AAV encoded (AAV8)	Hepatic expression	CNS storage reduced	MPS-IIIA mice	Ruzo et al., 2012 [173]
		Recombinant human HNS	IDDD	CNS storage reduced; Brain distribution	Cynomolgus monkeys	Chung et al., 2017 [174]
		IgG fusion	IP	CNS storage reduced	MPS-IIIA mice	Boado et al., 2018 [175]
MPS IIIB	α-*N*-acetylglucosamine 6-sulfatase	IgG fusion (HIR)	IV	Brain distribution	Rhesus Monkey	Boado et al., 2016 [110]
		Recombinant human NAGLU	ICV	CNS storage reduced; Increased enzyme activity	MPS IIIB mice	Kan et al., 2021 [176]
MPS IIIC	α-glucosaminidase acetyltransferase	AAV encoded (AAV-TT)	IC	Increased enzyme activity; CNS storage reduced;	MPS-IIIC mice	Tordo et al., 2018 [177]
				Decreased astrocytosis and lysosomal burden		
MPS IIID	*N*-acetylglucosamine 6-sulfatase	AAV encoded (AAV9)	ICV	Increased enzyme activity; CNS storage reduced; Improved lysosomal functionality	MPS-IIID mice	Roca et al., 2017 [178]
		Recombinant GNS	ICV	Increased enzyme activity; CNS storage reduced;	MPS-IIID mice	Wang et al., 2020 [179]
				rhGNS localized to lysosome		
MPS IVA	*N*-acetylgalactoasmine-6-sulfate sulfatase	Recombinant human GALNS	IV	CNS storage reduced	MPS-IVA mice	Tomatsu et al., 2008 [180]
		Recombinant human GALNS	IV	Brain distribution	WT mice	Álvarez et al., 2019 [181]
		(Nanostructured Lipid Carrier)				
MPS IVB	β-galactosidase	Recombinant human β-gal	ICV	Brain distribution; CNS storage reduced; Increased enzyme activity	MPS-IVB mice	Chen et al., 2020 [85]
MPS VI	*N*-acetylgalactosamine 4-sulfatase	Recombinant ARSB	IT	CNS storage reduced	MPS-VI cats	Auclair et al., 2012 [182]
MPS VII	β-Glucuronidase	Chemical	IV (high dose)	CNS storage reduced	MPS-VII mice	Huynh et al., 2012 [183]
		Recombinant, AAV encoded	IT	Increased enzyme activity; Reduction of lysosomal enlargement in neuroglia and Purkinje neurons	MPS-VII mice	Pagés et al., 2019 [184]
		(AAVrh10)				
		Recombinant human GUSB	IV	CNS storage reduced; Brain distribution	MPS-VII mice	Cadaoas et al., 2020 [20]
MPS IX	Hyaluronidase	No CNS involvement			MPS-IX mice	Martin et al., 2008 [185]

ETV: enzyme transport vehicle; RTB: plant lectin ricin B chain; IDDD: Intrathecal drug delivery device; IT: Intrathecal; IV: Intravenous; ICV: Intracerebroventricular; IN: Intranasal; IP: Intraperitoneal; IC: Intracranial

While most BBB delivery mechanisms that utilize antibody-enzyme conjugates rely on the Fab region of the antibody to bind and activate RMT, Ullman et al. demonstrated a novel antibody-enzyme scheme that utilized the Fc region of the antibody to bind TfR and trigger RMT [65]. Their compound was formed by conjugating the Fc domain of an IgG1 antibody to IDS, and they showed that the resulting fusion protein was able to decrease the pathological accumulation of GAGs in the brain as well as in peripheral tissues in a murine MPS II model. Interestingly, the monovalent binding of the Fc region allowed not only improved delivery of the enzyme, but also prevented the disruption of normal TfR trafficking that can be seen when using the bivalent Fab domains to trigger RMT.

A similar study performed by Arguello et al. showed that IDS fused to Fc domain with a moderate affinity to TfR had a greater delivery efficiency compared to high-affinity constructs in the brain of the MPS II mouse model [66]. Interestingly, their capillary depletion study of these constructs indicated that IDS fused to the Fc domain with TfR epitope distributed brain parenchyma, while the brain distribution of IDS fused to C-terminal of high affinity bivalent anti-TfR antibody was limited within the capillary fraction of the brain. These studies confirm the importance of TfR binding affinity for effective dissemination of the enzyme construct to treat abnormal CNS storage. In addition to TfR-mediated brain delivery, there are multiple studies using the insulin receptor intrinsically expressed in the brain endothelial cells to deliver lysosomal enzymes across the BBB. These studies include hexosaminidase (Tay-Sachs disease), palmitoyl-protein thioesterase-1 (Batten disease type 1), acid sphingomyelinase (Niemann-Pick type A and B), and β-galactosidase-1 (GM1-gangliosidosis) [60, 104]. These studies further open an avenue of RMT of lysosomal enzymes across the BBB.

While there are significant advances in RMT with bioengineered antibody-based delivery, other groups have explored conjugating enzymes to alternative compounds in order to cross the BBB. For example, Salvalaio et al. synthesized a poly(lactide-co-glycolide) (PLGA) copolymer nanoparticle to transport albumin labeled with FITC across the BBB as a preliminary test for large molecular weight compounds [105]. By coating the PLGA nanoparticle in a 7-amino acid long glycopeptide (g7), the group showed that the complex was able to transport FITC-labeled albumin across the BBB in both MPS I and MPS II mice models. In a follow-up study, this group tested the efficacy of the g7-PLGA complex in delivering IDS across the BBB to treat MPS II mice, which resulted in reduced GAG concentration in both brain and liver tissue [106]. The B subunit of the ribosome-inactivating toxin (RTB), a lectin, has emerged as another novel carrier for enzymes across the BBB [107]. RTB:alpha-L-iduronidase fusion proteins were made via a plant-based platform using *N. benthamiana* leaves, and tested in an MPS I mouse model, resulting in decreased GAG concentrations in brain parenchyma as well as improvements in murine neurocognition, as measured via the Barnes maze test.

Using a different modality which did not require a fusion protein, Cadaoas et al. tested the effect of sialylated recombinant human GUSB on crossing the BBB to treat MPS VII [20]. Interestingly, the increase in sialylation not only showed improved systemic half-life, brain distribution, and decreased GAG accumulation in a murine model of MPS VII. While the exact mechanism of the brain uptake of sialylated GUSB remains to be elucidated, this study suggests that extensive prolongation of plasma half-life increased the bioavailability of GUSB to the brain in an amount (1 ~ 3%) sufficient to normalize the abnormal GAG accumulation [20].

Using another approach to improve treatment efficacy, Cho et al. investigated the effect of enzyme dosage manipulation on the overall outcome rather than enzyme modification [108]. In the MPS II mouse model, the reduction of GAG concentration in the brain was dependent on the dose of intravenously administered IDS, yet this dose-dependent effect was only seen in younger mice that were administered therapy at 2 months of age instead of 8 months likely due to ineffective transport via down-regulated CI-MPRs at the adult BBB.

In addition to in vitro and murine studies, Boado et al. have tested the pharmacokinetics of lysosomal enzymes conjugated to the human insulin receptor in rhesus monkeys [109, 110]. The fusion protein made of alpha-*N*-acetylglucosaminidase combined with an IgG antibody against insulin receptor was shown to cross the BBB in a sufficient amount to treat GAG accumulation in the brain [109]. The pharmacokinetic profiles of iduronidase and iduronidase 2-sulfatease conjugated with human insulin receptor antibodies were compared between intravenous and subcutaneous administrations in Rhesus monkeys [110], and the results indicated that prolonged levels of the conjugates in the systemic circulation, enabling the longer availability for the uptake in the brain and periphery.

Overall, the brain uptake of lysosomal enzyme fused to human insulin antibody could be a great strategy for treating CNS storage. Also, these studies in primates helped to understand the systemic biodistribution of antibody fusion construct of lysosomal enzymes, which also encompassed the animal scale-up for further development of the therapeutic interventions in MPS patients.

## Clinical advancements in MPS therapies

### MPS I

While there have been many promising discoveries coming out of pre-clinical research, there have also been a number of meaningful advancements in the clinical world of MPS research (Table 5). An enzyme-antibody complex of clinical interest is Valanafusp alpha, which consists of the enzyme alpha-l-iduronidase conjugated to an IgG antibody, and was developed for the treatment of MPS I. In 2018 Pardridge et al. evaluated the pharmacokinetics of Valanafusp alpha in 18 patients, five adults and 13 children, with MPS I [111]. This study showed that plasma clearance of the drug was significantly greater in children when compared to adults. Furthermore, the elimination half-life of the enzyme-antibody conjugate, Valanafusp alpha, was shown to be similar to that of the unconjugated IUDA enzyme in children with MPS I [35]. In phase I and II trials, the same group showed that valanafusp alpha resulted in the improvement of cognitive and somatic symptoms associated with MPS I patients [111]. In this study, 11 patients between the ages of 2–15 years were treated with intravenous infusions of Valanafusp alpha at 1, 3, or 6 mg/kg for 52 weeks. Urinary GAGs decreased in all patients and an improvement was seen in the development quotient, an age-adjusted cognitive test which assesses a child's level of cognitive development. In addition to the neurocognitive improvement, the trial revealed a significant reduction in liver and spleen volumes as well as significant improvement in joint mobility suggesting that the enzyme was delivered to both the brain and peripheral organs.

**Table 5:** Summary of MPS clinical research

MPS type	Lysosomal enzyme	Modification	Administration	Findings	# of patients†	References
MPS I	α-l-iduronidase	Recombinant human IDUA, followed by Pentosan polysulphate (PPS)	ERT: IV PPS: SQ	↓ uGAGs*; Improved joint mobility and range of motion; Decreased pain	4	Hennermann et al., 2016 [186]
		Recombinant human IDUA, IgG fusion	IV	Stabilized developmental quotient, GM volume, & uGAGs; Improvement in joint mobility*; Reduced spleen and liver volume*	11	Giugliani et al., 2018 [111]
		IgG Fusion (HIR)	IV	Increased plasma clearance in children compared to adults*	5 adults 13 children	Pardridge et al., 2018 [35]
MPS II	Iduronidase-2-sulfate	Recombinant human I2S	IV	↓ uGAGs*; Improvements in endurance (6MWT)*	31	Sohn et al., 2013 [113]
			IDDD	> 70% ↓ CSF GAGs	12	Muenzer et al., 2016 [112]
		Recombinant human I2S with anti-human transferrin receptor antibody (JR-141)	IV	↓ CSF GAGs*; ↓ plasma and uGAGs	14	Okuyama et al., 2019 [115]
				↓ CSF GAGs*;↓ serum GAGs; Stabilized endurance	28	Okuyama et al., 2021 [36]
				↓ CSF GAGs* (2.0-mg/kg); Decreased liver and spleen volume; Improvement of neurocognition; Stabilized cortical GM volume	20	Giugliani et al., 2021 [116]
MPS IIIA	Heparan-*N*-sulfatase	Recombinant human HNS	IDDD	↓ CSF heparan sulfate; ↓ uGAGs	12	Jones et al., 2016 [117]
			IT	↓ CSF GAGs; ↓ uGAGs*	14	Wijburg et al., 2019 [118]
MPS IIIB	α-*N*-acetylglucosamine 6-sulfatase	Recombinant human NAGLU, AAV encoded (rAAV2/5)	IP	Enzyme activity found; Improved neurocognition	7	Tardieu et al., 2017 [187]
MPS IVA	*N*-acetylgalactoasmine-6-sulfate sulfatase	Recombinant Human GALNS	IV	↓ urinary Keratin Sulfate; Improved endurance (6MWT, 3MSC) and respiratory function	117	Hendriksz et al., 2015 [121]
				Improvements in exercise capacity, muscle strength, and pain	25	Burton et al., 2015 [120]
				Stabilized endurance, respiratory function, and ability to perform ADLs‡	20	Hendriksz et al., 2018 [37]
MPS VI	*N*-acetylgalactosamine 4-sulfatase	Recombinant human ARSB	IV	↓ uGAGs*; Improved 12MWT compared to placebo*	19	Harmatz et al., 2005 [38]
MPS VII	β-Glucuronidase	Recombinant human GUSB	IV	↓ uGAGs*; ≥ 1 improvement in Multi-Domain Responder Index category in 10/12 patients	12	Harmatz et al., 2018 [39]
				↓ uGAGs*	23	Qi et al., 2018 [123]
				↓ uGAGs	3	Cadaoas et al., 2020 [20]
				↓ uGAGs*; Reduction of fatigue; Stabilized visual acuity, joint mobility, fine motor skills	12	Wang et al., 2020 [122]
				↓ uGAGs*	3	Jones et al., 2021 [188]

IDDD: Intrathecal drug delivery device; IT: Intrathecal; IV: Intravenous; IP: Intraparenchymal; SQ: Subcutaneous

^†^Accounts for subjects receiving treatment

^‡^Does not have placebo control

^*^ < 0.05 significance

Of note, clinical trials for MPS IVB, IIIC, IIID, and X could not be located

### MPS II

Human MPS II treatments have also been explored using recombinant IDS which lowered the accumulation of GAGs in the peripheral organs [112, 113]. However, managing the CNS storage has remained as intravenous IDS administration was ineffective to treat CNS pathology [40, 114]. Okuyama et al. tested the safety of pabinafusp alfa, IDS conjugated to an anti-TfR antibody, in 14 patients [115]. Patients were intravenously administered doses of either 1 or 2 mg/kg/week for 4 weeks. Plasma and urine heparin sulfate and dermatan sulfate were decreased in both groups. Notably, GAGs in the cerebrospinal fluid (CSF) were significantly decreased and two patients experienced improvements in neurocognition. These findings suggest that this enzyme-antibody complex is effective at crossing the BBB and therefore has therapeutic potential as a means of addressing the central component of MPS II.

In a follow-up phase 2/3 open-label trial, the same group assessed the efficacy of 2.0 mg/kg/week pabinafusp alfa in 28 adolescent MPS II patients over the course of 52 weeks [36]. In addition to a decrease in the concentration of heparan sulfate in  the CSF, there were improvements in neurocognitive development in 21 out of 28 patients, as well as decreases in serum GAG concentrations and liver and spleen sizes. In a similar phase 2 trial in Brazil, pabinafusp alfa was given intravenously to 20 patients at doses of 1.0, 2.0, or 4.0 mg/kg/week for 26 weeks, and the dosage safety profile was evaluated showing that the 2.0 mg/kg dose had the most favorable safety versus efficacy ratio [116]. Another method that has been explored to overcome BBB is the intrathecal injection of idursulfase in MPS II patients. In a phase 1/2 study, Muenzer et al. gave intrathecal injections to 16 patients at doses of 0, 1, 10, or 30 mg/month for 6 months in addition to weekly intravenous injections of 0.5 mg/kg, which resulted in a 90% decrease in CSF GAGs in the 10 and 30 mg group as well as an 80% decrease in the 1 mg/kg group [112].

### MPS III

An example of direct administration of a replacement enzyme to the CNS in another subtype of MPS comes from a study conducted by Jones which investigates intrathecal injection as the route of enzyme infusion for the treatment of MPS IIIA. Jones et al. administered human recombination heparan-*N*-sulfatase intrathecally in 12 patients at doses of 10, 45, or 90 mg per month for 6 months in a phase 1/2 trial [117]. While there was a decline in CSF heparan sulfate concentrations, there was no significant difference in neurocognitive measures. More recently, a study was performed where 21 patients were randomized to groups of no treatment, or 45 mg of intrathecal heparan sulfate either every two weeks or four weeks. Though there was again a decrease in CSF heparan sulfate and urine GAGs, no changes between the treated and untreated groups were observed in terms of adaptive behavioral function or cortical gray matter volume [118]. Intravenous administration of *N*-acetyl-α-d-glucosaminidase to treat MPS IIIB has also been studied in an 11-patient trial of children below 12 years of age. While the treatment had minimal adverse effects, there was no clinically significant improvement in neurocognition [119]. Although the intrathecal approach to ERT is potentially valuable due to being able to avoid interacting with the complex transport system of the BBB, the lack of improvement in neurocognitive function in the results of these studies indicates that further adjustments are necessary before it is a viable option for treating the central component of MPS.

### MPS IV

ERT by elosulfase Alfa has been explored as a treatment for MPS IVA. In 2015, Burton et al. performed a phase 2, randomized, double-blind trial with 25 patients all above the age of 7 to test the safety and improvement in exercise capacity of weekly intravenous doses of either 2 mg/kg or 4 mg/kg elosulfase alfa for 25 weeks [120]. The results of the study showed no safety concerns at either dose as well as an improvement in the 3-min stair climb test (3MSCT). However, due to a limited sample size and the heterogeneous nature of MPS disease presentation, statistically significant changes were unable to be found in either group. In a separate MPS IVA phase 3 study, Hendriksz et al. compared the efficacy of a 24-week regimen with 2.0 mg/kg elosulfase alfa administered either weekly or every other week versus placebo [121]. Efficacy measures showed modest improvements in the 6-min walk test (6MWT), 3MSCT, maximal voluntary ventilation, and urine concentration of keratan sulfate compared to weekly treatments versus placebo or treatment every other week.

In a follow-up study, this group assessed the effects of the immunogenicity of elosulfase alfa on the treatment efficacy of elosulfase alfa in two sequential studies [37]. In the first open-label trial, 20 patients were given a weekly intravenous dose of 0.1, 1.0, or 2.0 mg/kg of elosulfase alfa for 36 weeks before an optional second trial of a weekly 1.0 mg/kg dose of elosulfase for up to 48 weeks. While all patients who participated in these two studies experienced at least one adverse event which ranged from mild to moderate severity, hypersensitivity reactions occurred in 25% of patients. Also, there was no correlation between the formation of antibodies against elosulfase alfa and the clinical outcomes in patients. However, the trial indicated a stabilization of endurance, respiratory function, and ability to perform activities of daily living. This is a notable study, however this trial lacked placebos so the results may not solely be attributed to the ERT by elosulfase alfa.

### MPS VI

With regards to MPS VI, ERT remains one of the primary therapies being studied to treat the symptoms associated with this MPS subtype. This is showcased by a phase 3, randomized, double-blind, placebo-controlled clinical trial conducted by Harmatz et al. investigating the potential of recombinant human arysulfatase B (rhASB) as a replacement enzyme to treat MPS VI. In this study, researchers found that patients receiving weekly intravenous rhASB infusions exhibited a significant increase in walking distance in the 12-min walk test and a significant decrease in urinary GAG levels compared to patients given a placebo [38]. The improvements in measures of systemic lysosomal clearance and endurance show that rhASB based ERT indeed has the potential to be an effective treatment for the peripheral component of MPS VI, however, a major drawback of this therapy is its inability to cross the BBB and the resulting neglect of the neurological aspect of the disease.

### MPS VII

ERTs for MPS VII have also been clinically explored in recent years using vestronidase alfa, a recombinant human GUSB. In a recent trial, 12 subjects were given 4 mg/kg of vestronidase alfa every 2 weeks starting at different time points across a 24-week period, which resulted in a significant reduction in urinary dermatan sulfate with at least one improvement in the Multi-Domain Responder Index category in 10 out of 12 patients [39]. In a follow-up publication, the same group extended the study for 144 weeks. Although seven subjects showed anti-enzyme antibodies, low urinary GAG concentrations were sustained. Additionally, there was a reduction in fatigue, but due to the lack of BBB transport, neurocognitive symptoms were not alleviated [122]. Qi et al. modeled the pharmacokinetics and pharmacodynamics of vestronidase alfa to suggest that a dose of 4 mg/kg every other week was the recommended dose [123].

Also, Cadaoas et al. reported that increased sialylation of vestronidase alfa reduced urinary GAG levels in a dose-dependent manner in phase 1/2 clinical trial, showing the further promising efficacy of ERT in MPS VII patients [20]. While these results clearly indicate a clinically meaningful benefit to this ERT in patients suffering from MPS VII, the lack of improvement in cognitive measures may highlight the need to further develop the capability of exogenous replacement enzymes to reverse neurological symptoms such as mental retardation.

## ERT concerns

Additional concerns for ERT surround its immunogenicity as well as the safety profile including the biodistribution of the bioengineered therapeutic agents throughout the body [62]. Improving the safety profile of ERT is an ongoing field of research with an emphasis placed on optimizing antibody design [124]. As discussed by Kishnani et al., ERT can elicit an antibody-mediated immune response resulting in loss of activity or enzyme removal; thus patients receiving ERT should routinely have antibody testing administered to monitor for potential hypersensitivity complications [37, 125]. Though there is a lack of standardization in the reporting and analysis of infusion-related reactions, Doessegger and Banholzer propose a methodology for grading anaphylactic symptoms and monitoring for reactions 24 h after infusion [126]. Ultimately, it is our desire to develop well-tolerated and demonstrated no toxicity at clinical dose.

## Conclusion

The delivery of lysosomal enzymes across the BBB to treat neurological symptoms in patients with MPSs remains an open challenge. Though there have been novel advances in increasing the delivery of enzymes across brain endothelial cells via transcytosis, the clinical efficacy of these methods has not been definitively shown across all MPS subtypes. Fusion proteins made by conjugating enzymes to antibodies offer a promising route for CNS delivery with some clinical evidence showing limited efficacy; however, there is still significant work required to fully treat the neurocognitive symptoms found in MPS patients. Additionally, the immunogenicity of these fusion proteins should be further studied to determine the possible long-term effects of the immune responses generated against ERT.

## Data Availability

Not applicable.
